# Impact of treatment with orthodontic aligners on the oral health-related quality of life

**DOI:** 10.1186/s12903-024-04183-z

**Published:** 2024-04-05

**Authors:** Gabriela Luiza Nunes Souza, Esdras de Campos França, Marcelo de Araújo Lombardi, Giselle Cabral da Costa, Najara Barbosa da Rocha, Lucas Guimarães Abreu

**Affiliations:** 1 Department of Pediatric Dentistry, School of Dentistry, Universidade Federal de Minas Gerais, Belo Horizonte, Brazil; 2 Associação Brasileira de Odontologia, Belo Horizonte, Brazil; 3 Department of Social and Preventive Dentistry, School of Dentistry, Universidade Federal de Minas Gerais, Belo Horizonte, Brazil; 4 Department of Pediatric Dentistry, School of Dentistry, Universidade Federal de Minas Gerais, Av. Antônio Carlos, Pampulha, Belo Horizonte, 6627 MG Brazil

**Keywords:** Quality of life, Orthodontics, Epidemiology, Aligners

## Abstract

**Background:**

There is an increasing interest in information on the effects of orthodontic aligners on the oral health-related quality of life (OHRQoL) of people.

**Aim:**

To compare the impact of orthodontic aligners versus conventional fixed appliances on OHRQoL, using a validated tool and controlling for sociodemographic and clinical variables.

**Method:**

Sixty-one individuals participated in this study. Group 1 (G1) consisted of 33 individuals under treatment with orthodontic aligners and Group 2 (G2) comprised 28 individuals under treatment with conventional fixed appliances. OHRQoL was evaluated with the Oral Health Impact Profile (OHIP-14) in which 14 items are distributed across seven dimensions: functional limitation, physical pain, psychological discomfort, physical disability, psychological disability, social disability, and handicap. The higher the score, the more negative is the perception of the individual regarding his/her OHRQoL. Descriptive statistics, Mann-Whitney test, and Poisson regression were performed. Effect Size (ES) and Minimal Clinically Important Difference (MCID) were also determined.

**Results:**

Participants’ mean age was 30.69 years. Individuals in G1 had a significantly lower score for physical pain and the total score of OHIP-14 compared to individuals in G2 (*p* < 0.05). The ES was large (ES = 0.74) for physical pain and moderate (ES = 0.46) for the total score. The ES was moderate for physical disability (ES = 0.50). The difference between groups for physical pain (1.30) and for physical disability (0.90) was greater than the MCID (0.87 and 0.88, respectively). Poisson regression showed that G2 individuals showed a score for physical pain 1.39 times higher than those of G1 in the adjusted model (OR = 1.39, [1.03–1.89], *p* = 0.031).

**Conclusion:**

Those under treatment with orthodontic aligners have a more positive perception of OHRQoL compared to those wearing fixed appliances.

## Introduction

The term Oral Health-Related Quality of Life (OHRQoL) pertains to the impact of oral health outcomes on daily routine that are relevant to patients and people, with such impact being of sufficient magnitude, either in terms of frequency, severity, or duration, to have an effect on the perceptions of individuals with respect to their well-being [1]. Unlike normative clinical indicators, OHRQoL measures strive to encompass the physical, functional, and psychosocial consequences of oral diseases and their treatments from the perspective of the individuals themselves, regardless of age (children, adults, or the elderly) [2]. It is widely acknowledged that malocclusion, for instance, has a detrimental effect on OHRQoL, particularly with substantial repercussions on psychosocial aspects [3, 4]. The literature also recognizes that sociodemographic factors, such as sex, age [5], and household income [6] may have an influence on OHRQoL.

Individuals with established occlusal changes often seek orthodontic services to enhance their dental aesthetics [7]. Numerous studies in the literature delve into the impact of orthodontic treatment with conventional fixed appliances on OHRQoL [4]. At the initiation of orthodontic treatment, wearing fixed appliances is known to have a negative impact on OHRQoL, potentially leading to physical problems such as pain and discomfort, along with functional issues like eating difficulties [8]. In the initial phases of treatment, individuals may also experience anxiety [9]. Conversely, in more advanced stages [10] or post-completion of the treatment [11], positive effects become evident, manifesting as improvements in both emotional and social well-being [12].

Some prospective orthodontic patients decline conventional fixed appliances due to challenges in mastication and device maintenance. As an alternative, orthodontic aligners have gained prominence in clinical practice for correcting malocclusion. However, evidence regarding the impact of wearing orthodontic aligners on OHRQoL compared to treatment with conventional fixed appliances is limited [13]. Recent systematic reviews have shown that only a limited number of studies have compared the OHRQoL of individuals wearing aligners and those undergoing orthodontic treatment with fixed appliances, using tools that had been submitted to formal psychometric validation [14–16]. Moreover, studies deploying regression analysis to compare the OHRQoL of wearers of these two orthodontic devices while controlling for confounding variables are also scarce in literature [17]. The evaluations in most studies are restricted to bivariate analysis associating quality of life and the type of device worn [18, 19]. Therefore, the objective of this study was to compare the impact of wearing orthodontic aligners with wearing conventional fixed appliances on OHRQoL, using a validated tool and controlling for sociodemographic and clinical variables.

## Methods

### Ethical issues

The Ethics Committee of the Federal University of Minas Gerais (CAAE- 39216920.0.0000.5149) approved this study. The right to refuse to participate in the study was guaranteed to the invited individuals. For those who agreed to participate, written consent was provided prior to data collection. Individuals who were 18 years or older and parents/guardians of those younger than 18 signed the Informed Consent Form (ICF). Individuals younger than 18 years signed the Free and Informed Assent Form (FIAF).

### Study design, participants, location, and eligibility criteria

A cross-sectional study was conducted. The sample consisted of 61 individuals undergoing orthodontic treatment in the Graduate Program in Orthodontics at Associação Brasileira de Odontologia (Brazilian Dental Association), Belo Horizonte, Brazil. Treatments were conducted by Graduate students. Individuals with cognitive disorders or other disorders reported by themselves or their parents/guardians and those with craniofacial anomalies were excluded from the study. During the assessment for eligibility, individuals were queried about formal diagnosis of cognitive disorders. Additionally, an examination was conducted to evaluate the presence of any craniofacial alterations. The reporting of this article followed the guidelines of STROBE initiative (Strengthening the Reporting of Observational Studies in Epidemiology) [20].

### Study variables

#### Dependent variable

oral health-related quality of life (OHRQoL).

The impact of an orthodontic treatment on OHRQoL was assessed with the Oral Health Impact Profile (OHIP-14) instrument. In its original version, the OHIP consists of 49 questions [21]. In 1997, a short form of the tool containing 14 questions was designed [22]. The 14 questions are distributed across seven dimensions: functional limitation, physical pain, psychological discomfort, physical disability, psychological disability, social disability, and handicap. Answers are given following a numerical scale: 0 = never, 1 = rarely, 2 = sometimes, 3 = often, and 4 = always. The score of each dimension ranges from 0 to 8. The sum of the answers of the 14 questions makes up the total score of the OHIP-14, which ranges from 0 to 56. The higher the score, the more negative is the perception of the evaluated individual regarding his/her OHRQoL [22]. The OHIP-14 has already been translated and validated into several languages, including Brazilian Portuguese [23].

#### Independent variable: type of orthodontic device

The 61 participants were divided into two groups. Group 1 (G1) consisted of individuals undergoing orthodontic treatment with aligners (Invisalign®). Group 2 (G2) consisted of individuals undergoing orthodontic treatment with conventional fixed appliances (Morelli® 0.022’’).

#### Confounding variables: sex, age, family income, and stage of treatment

The following confounding variables were assessed: individuals’ sex (male/female) and age (in years), and monthly family income. The monthly family income was assessed according to the Brazilian minimum wage at the time of data collection and established by adding up the monthly income of all economically active family members. This variable was dichotomized into individuals whose families had a monthly income ≤ 3 minimum wages and individuals whose families had a monthly income > 3 minimum wages. Data on the duration of orthodontic treatment from treatment onset until data collection (in months) were collected.

### Pilot study

A pilot study to evaluate the data collection strategy was conducted with individuals who were not included in the main study. The individuals had no difficulties in answering the OHIP-14 instrument. The researcher filled out a form to gather data on sociodemographic characteristics (sex, age, and family income) and clinical characteristics (type of orthodontic device worn and stage of treatment/duration of treatment in months from treatment onset until data collection). No change in data collection strategy was required.

### Statistical analysis

Statistical analysis was performed through the Statistical Package for Social Science (SPSS, version 25.0, IBM Inc., Armonk, USA). First, a descriptive analysis of the data was performed. The Kolmogorov-Smirnov test demonstrated that the OHIP-14 dimensions’ scores and the total score had a non-normal distribution. Bivariate analysis with the Mann-Whitney test compared the dimension scores and the OHIP-14 total score between G1 and G2. The differences between G1 and G2 for the OHIP-14 dimensions and the total score and their respective 95% confidence intervals (CI) were calculated. The effect size (ES) of these differences and their respective 95% CIs were also determined. Values ​​close to 0.2 indicated a small ES, values ​​close to 0.5 indicated a moderate ES and values ​​close to 0.8 indicated a large ES [24]. When comparing groups, the Minimal Clinically Important Difference (MCID) was determined by multiplying the standard deviation of dimensions scores and the total score of OHIP-14 of the entire sample by 0.5 [25]. Finally, Poisson regression was performed comparing G1 and G2 for the scores that exhibited a statistically significant difference between groups in the bivariate analysis. The model was controlled for the variables sex and age of individuals, duration of orthodontic treatment, and monthly family income. In all analyses, the statistical significance level was set at *p* < 0.05.

## Results

G1 was composed of 33 individuals (54.1%) and G2 was composed of 28 individuals (45.9%). Participants’ age ranged from 11 to 54 years old (mean = 30.69 ± 11.06). Table 1 shows the demographic characteristics of the participants.

**Table 1:** Demographic characteristics of the sample

Variables	G1: Orthodontic aligner	G2: Fixed appliance
**Age (years)**		
Mean Median Standard deviation Minimum Maximum	33.73 32.00 8.76 21 54	27.11 23.50 12.50 11 54
**Sex - N (%)**		
Female Male	16 (48.5%) 17 (51.5%)	17 (60.7%) 11 (39.3%)
**Income - N (%)**		
≤3 minimum wages >3 minimum wages	05 (15.2%) 28 (84.8%)	20 (71.4%) 08 (28.6%)
**Treatment time (months)**		
Mean Median Standard deviation Minimum Maximum	11.52 10.00 6.85 2 24	22.39 22.00 14.45 2 60

Individuals in G1 had a significantly lower score for the physical pain dimension (*p* = 0.004) and for the total score of OHIP-14 (*p* = 0.023) compared to individuals in G2. The ES for physical pain was large (ES = 0.74) and for the total score of OHIP-14, the ES was moderate (ES = 0.46). A moderate ES was also observed for the physical disability dimension (ES = 0.50). The differences between groups for the physical dimension (1.30) and for the physical disability dimension (0.90) were greater than the MCID (0.87 and 0.88, respectively). The results of the bivariate analysis are displayed in Table 2.

**Table 2:** Bivariate analysis comparing the OHIP-14 dimensions and the total score of OHIP-14 between wearers of orthodontic aligners (G1) and wearers of conventional fixed appliance (G2)

	G1 Median (Min-Max) Mean (SD)	G2 Median (Min-Max) Mean (SD)	*p* value^*^	Difference between G1 and G2 (95% CI)	Effect Size (95% CI)	MCID
**Functional limitation**	1.00 (0–5) 1.06 (1.36)	1.00 (0–5) 0.89 (1.16)	0.745	0.168 (-0.490–0.825)	0.13 (-0.38–0.64)	0.635
**Physical** **pain**	3.00 (0–5) 2.73 (1.56)	4.00 (0–7) 4.04 (1.73)	0.004	-1.308 (-2.154 - -0.463)	0.74 (0.23–1.25)	0.879
**Psychological discomfort**	2.00 (0–8) 2.58 (2.43)	3.00 (0–7) 3.18 (2.03)	0.212	-0.603 (-1.766–0.561)	0.26 (-0.25–0.77)	1.132
**Physical disability**	0.00 (0–6) 0.85 (1.37)	1.00 (0–7) 1.75 (2.06)	0.058	-0.902 (-1.823–0.020)	0.50 (-0.01–1.01)	0.884
**Psychological disability**	1.00 (0–8) 1.61 (1.88)	2.50 (0–8) 2.50 (2.15)	0.076	-0.894 (-1.928–0.141)	0.43 (-0.08–0.94)	1.022
**Social disability**	0.00 (0–5) 0.64 (1.14)	1.00 (0–6) 1.18 (1.51)	0.080	-0.542 (-1.224–0.140)	0.40 (-0.11–0.91)	0.671
**Handicap**	0.00 (0–8) 0.76 (1.54)	0.00 (0–4) 0.64 (1.31)	0.494	0.115 (-0.626–0.856)	0.08 (-0.43–0.59)	0.715
**Total score**	9.00 (0–39) 10.21 (8.50)	12.00 (0–38) 14.18 (8.01)	0.023	-3.966 (-8.225 - -0.292)	0.46 (-0.05–0.97)	4.452

G1 = Group 1 (individuals wearing orthodontic aligners), G2 = Group 2 (individuals wearing conventional fixed appliances), Min = Minimum, Max = Maximum,

SD = Standard Deviation, CI = Confidence interval, MCID = Minimal clinically important difference, ^*^Mann-Whitney test. Statistically significant at *p* < 0.05

Poisson regression was performed for the physical pain dimension and the total score of OHIP-14, the two scores that exhibited a statistically significant difference between groups in the bivariate analysis. The adjusted results of the Poisson regression showed that individuals in G2 had a physical pain dimension score 1.39 times higher than individuals in G1 in the adjusted model (OR = 1.39, [1.03–1.89], *p* = 0.031) (Table 3).

**Table 3:** Poisson regression comparing the impact of orthodontic aligners (G1) and conventional fixed appliance (G2) on physical pain and the total score of OHIP-14

	Physical pain				Total score			
	Non-adjusted		Adjusted		Non-adjusted		Adjusted	
	OR (95% CI)	*p* value^*^	OR (95% CI)	*p* value^*^	OR (95% CI)	*p* value^*^	OR (95% CI)	*p* value^*^
**Type of orthodontic device**								
G1 G2	1 1.48 (1.15–1.89)	0.002	1 1.39 (1.03–1.89)	0.031	1 1.38 (0.98–1.96)	0.064	1 1.24 (0.68–2.25)	0.472
**Sex**								
Female Male	1 1.10 (0.86–1.42)	0.423	1 1.01 (0.78–1.30)	0.934	1 1.00 (0.71–1.40)	0.998	1 1.05 (0.73–1.53)	0.766
**Family income**								
≤3 MW >3 MW	1 0.70 (0.54–0.89)	0.005	1 0.75 (1.02–0.54)	0.076	1 0.70 (0.50–0.99)	0.047	1 0.75 (0.42–1.36)	0.356
**Age (years)**								
	0.99 (0.98–1.01)	0.812	1.01 (0.99–1.02)	0.582	0.99 (0.97–1.01)	0.155	0.99 (0.98–1.01)	0.356
**Treatment time (months)**								
	1.01 (0.99–1.02)	0.473	0.99 (0.98–1.01)	0.114	1.01 (0.99–1.02)	0.423	0.99 (0.97–1.01)	0.399

G1 = Group 1 (individuals wearing orthodontic aligners), G2 = Group 2 (individuals wearing conventional fixed appliances)

OR = Odds ratio, CI = Confidence Interval, ^*^Poisson regression. Statistically significant at *p* < 0.05

MW = Minimum wage

## Discussion

The results of this study show that individuals undergoing treatment with orthodontic aligners have a more positive perception of physical pain and OHRQoL (total score of OHIP-14) compared to individuals undergoing treatment with conventional fixed appliances, with large and moderate ES, respectively. For the physical pain dimension, the result was confirmed in the regression model. The ES was also moderate for physical disability. Mean differences between groups were higher than the MCID for physical pain and physical disability. The results of our studies align with the findings of other studies [26–28], which also demonstrated that, during orthodontic treatment, wearers of aligners have an improved perception of their OHRQoL in comparison to fixed appliance wearers, with the main positive effects being upon the physical pain and physical disability dimensions, as well as the overall score.

Pain is a subjective response influenced by various factors, including age, gender, individual pain perception (pain threshold), emotional state, stress levels, and the force applied during activation of the orthodontic device. Cultural differences and past experiences with pain also play a role [29, 30]. The complaint of pain is a common outcome during orthodontic treatment and is a significant factor contributing to treatment drop-out and discontinuation [31]. Our results and findings of studies [26, 27] that also used the OHIP-14 to compare the OHRQoL between wearers of orthodontic aligner and wearers of fixed appliances indicate that the choice of orthodontic treatment can impact the perception of pain and discomfort caused by the orthodontic device itself. Individuals undergoing orthodontic treatment with aligners appear to report diminished pain scores compared to those undergoing treatment with fixed appliances [32]. Consequently, this choice can influence a patient’s adherence to treatment and cooperation throughout the entire orthodontic therapy.

While orthodontic brackets boast rounded and smooth surfaces, their wings and hooks come into contact with the lips and buccal mucosa, posing the potential for irritation and soft tissue wounds, and consequently leading to pain, mainly at the earlier stages of orthodontic therapy [33]. In contrast, orthodontic aligners, being tray-based, lack defined wings and hooks, thereby reducing the likelihood of irritation and wounds in the buccal mucosa [34]. Additionally, the forces exerted by fixed appliances can vary in magnitude, depending on the orthodontic forces applied by the orthodontist. This stands in contrast to the more precise and customized forces delivered by orthodontic aligners, whose planning is conducted digitally [34].

The comparison between orthodontic aligners and conventional fixed appliances did not reveal a statistically significant difference in terms of physical disability among the groups. However, the ES between these groups for this dimension was found to be moderate. The ES serves as a measure to assess the magnitude of the effect between the two therapies, irrespective of the presence of statistically significant results [35]. Calculating the ES proves crucial in studies comparing therapies [36], aiding clinicians in interpreting study results [37]. The current study’s findings indicate that individuals wearing orthodontic aligners tend to perceive physical disability less negatively than those wearing conventional fixed appliances, equally to what has been reported elsewhere [27, 28]. The main differences occur in daily eating and chewing performance [38]. This result could be attributed to the smaller size of aligners and the option to remove them during treatment [34], the latter, a feature unavailable to wearers of fixed appliances.

The interpretation of differences in the impact on OHRQoL between treatments has garnered increased interest among clinicians and researchers in recent years. This interpretation involves establishing the MCID and assessing whether statistical differences in the comparison between two therapies can be translated into clinical significance—meaning a minimum level of alteration that is both real and perceptible to the patient [39]. In our results, the mean differences between groups surpassed the MCID for physical pain and physical disability. Consequently, these differences between groups may be considered clinically relevant alterations, affirming the more positive impact of orthodontic aligners on physical issues compared to conventional fixed appliances [40]. Determining the MCID is crucial, as it signifies a change that the patient would find significant and representative [25].

This study bears the limitations inherent to a cross-sectional design, preventing the establishment of a causal relationship [41] between the exposure (type of device worn) and the outcome (OHRQoL). Another constraint lies in the recruitment process, as participants were sourced from a Graduate Program in Orthodontics, leading to treatment by different dentists. Consequently, there was a lack of standardization in the care provided by the orthodontists, despite all patients attending the same orthodontic service.

The outcomes of studies on OHRQoL are crucial for clinicians, aiding in the comprehension of the physical and functional consequences of orthodontic treatment and its effects on individuals’ well-being [42]. This information is particularly significant for orthodontists when recommending the type of appliance, as such a choice can influence patients’ perceptions of their daily lives and well-being. Consequently, it may impact levels of treatment discipline and the likelihood of drop-outs. Additionally, elucidating the effects of orthodontic therapy is critical for decision-makers, enabling them to enhance the quality of orthodontic care services. Future longitudinal studies should be conducted to determine whether a cause-and-effect relationship exists between the wearing of orthodontic aligners and their impact on patients’ OHRQoL.

## Conclusion

Wearers of orthodontic aligners have a more positive perception regarding their OHRQoL compared to wearers of conventional fixed appliances, particularly in terms of physical pain and physical disability.

## Data Availability

The data that support the findings of this study are available from the corresponding author upon reasonable request.

## Abbreviations

OHRQoL: Oral health-related quality of life
OHIP: Oral Health Impact Profile
ES: Effect Size
MCID: Minimal clinically important difference
